# A Case of Uterine Hemorrhage Three Weeks after Delivery

**Published:** 1859-01

**Authors:** G. A. Kunkler

**Affiliations:** Madison, Indiana


ART. IV.—*A Case of Uterine Hemorrhage Three Weeks After Delivery.* By G. A. KUNKLER, M.D., of Madison, Indiana.

THE occurrence of uterine hemorrhage several days or weeks after delivery is such an unusual accident that I have considered the following case worthy of particular notice: Mrs. A——, aged about thirty, the mother of several children, and possessed of a strong, robust constitution, was delivered, during May, 1855, of a large and healthy child. The labor had been an easy one of about eight or nine hours' duration, the placenta following in the course of fifteen minutes. The patient kept her bed for about five days, and did well, no unusual symptoms of any kind presenting themselves.

On the twentieth day after her confinement I was hurriedly called to see her, late in the evening, and was informed that for several hours blood had been escaping almost continually from the vagina. She was already very much prostrated from the hemorrhage; the countenance pale and anxious; the pulse exceedingly weak, and making over 115 beats per minute. The bed upon which she was lying was perfectly saturated with blood, and her extremities cool and clammy. The discharge was still quite profuse when I arrived; the amount lost could hardly be estimated, but must have been great, to have reduced so strong and healthy a person to such an extent.

Upon examination the vagina was found partially filled with several large coagula, and the os uteri dilated, readily admitting two fingers; the uterus itself was rather larger and softer than is usual at this period. A bladder filled with pounded ice was applied to the abdomen, followed by compresses dipped in ice water; three drachms of the wine of ergot were given at once, to be repeated in fifteen minutes, and absolute quiet and rest in the horizontal posture enjoined. A moderate contraction of the uterus followed; and upon the frequent exhibition of powdered ergot and tannin the discharge gradually and steadily diminished in quantity, until the fourth day, when it ceased entirely. To relieve the constipation, an enema of cold water with sulphate of magnesia was occasionally administered. With the aid of a nourishing and proper diet the patient convalesced rapidly. She was kept at perfect rest for about ten days afterwards, and has since been confined again.

I was unable to discover any positive and satisfactory cause of the hemorrhage in this case. The patient had not exerted herself to such an extent, at any time, that it could be ascribed to over-exertion; she stated, however, that during that day she had been in a violent state of mental excitement on account of some family difficulty; this is the only cause to which I could attribute it. The hemorrhage could not have resulted from a retention of a portion of the placenta, as the physician, Dr. Gräeter, who attended her in her confinement was positive that everything had passed, and besides the patient had no discharge of any kind after the seventh day, until the time I was called in.

During the month of July, 1857, while attending physician to the County Asylum, a case somewhat similar to the above came under my care. In this case the hemorrhage was caused by the retention of putrid coagula. The woman, an inmate of the asylum, was delivered by myself of a large, healthy child. The labor was rather tedious, owing to inertia of the uterus, requiring the free exhibition of ergot. The placenta followed about half an hour after the birth of the child. The lochia were very profuse, but the discharge had nearly disappeared on the sixth day after her confinement; but on the eighth day, while walking across the room, a profuse hemorrhage appeared. On making a vaginal examination, the os uteri was found dilated, and on passing two fingers into the uterus, several large and fetid clots were removed; the hemorrhage then ceased. The free use of ergot, a firm bandage around the abdomen, and injections into the vagina, soon restored her completely.

Cases like the above are very annoying, especially to the younger practitioner, from the fact that the hemorrhage is frequently regarded as *prima facie* evidence that there was some mismanagement in the third stage of labor, or want of proper care in the subsequent treatment; and it is not impossible that the prevalence of this opinion may, in some measure, account for the silence of authors upon this subject.

Although secondary hemorrhage is very often the result of ignorance and rashness, yet no reasonable person would or could maintain that it was invariably the case, any more than it could be said of any of the various casualties incident to labor, such as convulsions, ruptured uterus, etc.

In the *New York Journal of Medicine* for September, 1850, Dr. Ferguson has reported an interesting case of hemorrhage two weeks after delivery; and in the *New York Annalist*, October, 1847, Dr. Stimson has reported a case, where hemorrhage occurred on the tenth day subsequent to parturition.

